# Enhancing 2-Ketogluconate Production of *Pseudomonas plecoglossicida* JUIM01 by Maintaining the Carbon Catabolite Repression of 2-Ketogluconate Metabolism

**DOI:** 10.3390/molecules23102629

**Published:** 2018-10-13

**Authors:** Wenjing Sun, Tjahjasari Alexander, Zaiwei Man, Fangfang Xiao, Fengjie Cui, Xianghui Qi

**Affiliations:** 1 School of Food and Biological Engineering, Jiangsu University, Zhenjiang 212013, China; 1000003523@ujs.edu.cn (T.A.); 1000004769@ujs.edu.cn (F.X.); 1000003286@ujs.edu.cn (F.C.); 1000003420@ujs.edu.cn (X.Q.); 2 Parchn Sodium Isovitamin C Co. Ltd., Dexing, 334221, China

**Keywords:** *Pseudomonas plecoglossicida*, 2-ketogluconate consumption, transcription analysis, carbon catabolite repression, glucose supply

## Abstract

2-Ketogluconate (2KGA) is an organic acid that is important for pharmaceutical, cosmetic, and environmental applications. *Pseudomonas plecoglossicida* JUIM01 strain is an important industrial 2KGA producer in China. In this paper, we found that *P. plecoglossicida* JUIM01 could convert glucose to 2KGA extracellularly, and the formed 2KGA was subsequently consumed after glucose was exhausted during the fermentation process. Experiments of glucose and 2KGA supplementation during fermentation process revealed that, only when glucose was exhausted, the strain started to consume the product 2KGA. Then, the mechanism of this phenomenon was investigated at transcription and protein levels, and the results indicated that *P. plecoglossicida* JUIM01 possesses carbon catabolite repression of 2KGA metabolism by glucose. Next, increasing the supply of glucose could attenuate 2KGA consumption and enhance the 2KGA yield from glucose. Finally, fed-batch fermentation of *P. plecoglossicida* JUIM01 resulted in 205.67 g/L of 2KGA with a productivity of 6.86 g/L/h and yield of 0.953 g/g glucose. These results can provide references for the industrial fermentation production of 2KGA and other fermentation products.

## 1. Introduction

2-Ketogluconate (2KGA) is an organic acid that is important for pharmaceutical, cosmetic, and environmental applications. It is a key intermediate in industrial manufacture of erythorbic acid (D-isoascorbic acid, a stereoisomer of ascorbic acid), an antioxidant widely used in food industry. Currently, microbial fermentation is the common method for 2KGA production. Microorganisms from genera *Gluconobacter*, *Pseudogluconobacter*, *Pseudomonas*, and *Klebsiella* have been used for 2KGA production. The strains *Pseudomonas fluorescens* AR4 and A46, and *Pseudomonas plecoglossicida* JUIM01, were screened by our group, and are used for industrial production of 2KGA in most Chinese erythorbic acid companies [1,2,3,4,5,6,7]. *P. fluorescens* AR4 strain could produce 135 g/L of 2KGA under optimized conditions during batch fermentation, and this strain is suitable for the production of commercially acceptable levels of 2KGA in semi-continuous culture [3]. Metabolically engineered *Gluconobacter suboxydans* could produce 72.3 g/L of 2KGA with a yield of 0.723 g/g glucose [8]. Under optimum conditions, *Klebsiella pneumoniae* could produce 186 g/L of 2KGA with a yield of 1.05 g/g glucose [6]. Using biotransformation method, 60 g/L resting cells (wet weight) of recombinant *Gluconobacter oxydans* strain could produce 321 g/L of 2KGA from glucose [9].

**Figure 1:**
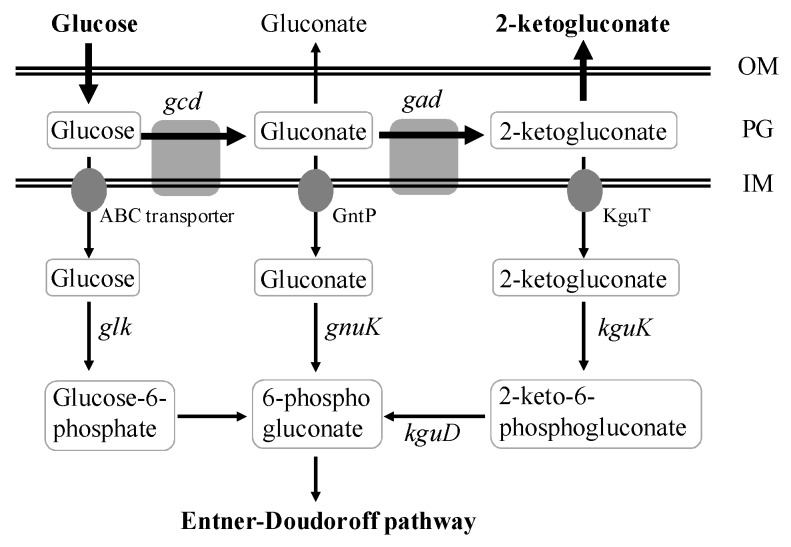
Schematic representation of 2-ketogluconate (2KGA) biosynthesis and catabolism pathways in *Pseudomonas*. OM, outer membrane; PG, periplasmic space; IM, inner membrane; ABC transporter, ATP-binding cassette transporter; GntP, gluconate transporter; KguT, 2-ketogluconate transporter. Genes: *gcd* encoding glucose dehydrogenase, *gad* encoding gluconate 2-dehydrogenase, *kguK* encoding 2-ketogluconate kinase, *kguD* encoding 2-keto-6-phosphogluconate reductase, *glk* encoding glucokinase, *gnuK* encoding gluconate kinase.

The bacteria of genus *Pseudomonas* metabolize glucose almost exclusively by Entner–Doudoroff (ED) pathway (Figure 1) [10,11,12]. The extracellular glucose can be converted to gluconate and 2KGA by two consecutive periplasmic oxidation reactions catalyzed by glucose dehydrogenase and gluconate dehydrogenase. Meanwhile, the intracellular glucose, gluconate, and 2KGA are converted to 6-phosphogluconate, and further channeled into the ED pathway [10,11,13,14]. For *P. putida* KT2440 strain, more than 80% of the glucose influx is channeled through the periplasmic oxidation pathway via gluconate or 2KGA and only a minor fraction through glucose-6-phosphate, and this strain can also use gluconate or 2KGA as the sole carbon source [11]. *P. fluorescens* can convert most glucose to gluconate, and 2KGA, the formed gluconate, and 2KGA are subsequently assimilated by the strain after glucose depletion [13].

Free-living bacteria usually have a versatile metabolism that allows the use of many different compounds as a source of carbon and energy. Generally, bacteria cells can selectively assimilate a preferred compound among a mixture of several potential carbon sources, and this process is carbon catabolite repression. Catabolite repression is a mechanism preventing transcriptional expression of genes required for the degradation of less-preferred substrate in the presence of the preferred substrate [15,16]. *Pseudomonas* are ubiquitous bacteria, and a wide range of compounds can be assimilated by them. The most unusual is that the preferred carbon sources for *Pseudomonas* are some organic acids or amino acids, rather than glucose. For example, in the presence of succinate and glucose, the expression of enzymes in the *P. aeruginosa* central pathway for glucose catabolism is repressed until succinate is consumed [15]. When *P. putida* KT2440 strain is cultured with succinate, glucose, gluconate, or 2KGA as the sole carbon source, succinate promotes the fastest growth, followed by gluconate and then by glucose, and 2KGA leads to the slowest growth. Thus, succinate is the most preferred substrate, and 2KGA is the least preferred substrate [11]. Glucose is used in preference to phenylacetic acid (PAA) when *P. putida* KT2440 strain is cultured in the presence of glucose and PAA, and the PAA catabolic genes are repressed by the presence of glucose [17]. However, for *P. putida* CSV86 strain, metabolic and transport studies demonstrate that glucose metabolism is suppressed when this strain is grown on aromatic compounds or organic acids [18]. Until now, there is almost no detailed research on the carbon catabolite repression of 2KGA metabolism by glucose in *Pseudomonas* strain.

*P. plecoglossicida* JUIM01 strain can synthesis 2KGA by using glucose as the substrate and currently used for erythorbic acid production in China [7]. In addition, there is no research on the relationship between glucose and 2KGA metabolism in *P. plecoglossicida* strain. In this study, we found that *P. plecoglossicida* JUIM01 could convert glucose to 2KGA extracellularly, and the formed 2KGA was subsequently consumed during later growth phase. Then, the mechanism of this phenomenon was investigated at transcription and protein levels. Finally, the consumption of 2KGA was attenuated by supplementation of sufficient glucose, and the 2KGA yield from glucose reached a high level.

## 2. Results and Discussion

### 2.1. 2KGA Production and Consumption by P. plecoglossicida JUIM01 at Low Initial Glucose Concentration

As mentioned above, *P. plecoglossicida* JUIM01 strain can synthesis 2KGA by using glucose as the substrate, and is an important industrial 2KGA producer in China [7]. In addition, some *Pseudomonas* strains can use not only glucose, but also 2KGA as the sole carbon source for cell growth [11,13]. In order to analysis the relationship between glucose and 2KGA metabolism in *P. plecoglossicida*, strain cultivation experiments with low glucose concentration in culture broth were performed.

**Figure 2:**
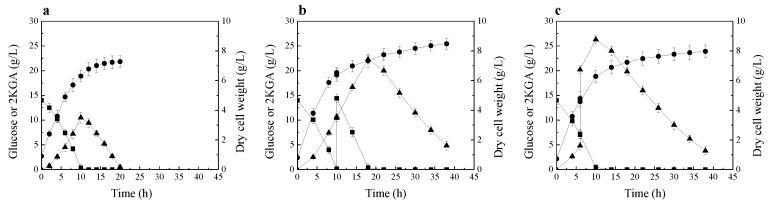
2KGA production and consumption by *P. plecoglossicida* JUIM01 at low initial glucose concentration. (**a**) 2KGA fermentation without glucose and 2KGA supplementation; (**b**) Glucose supplementation experiment; (**c**) 2KGA supplementation experiment. Signal denotes: filled triangle—2KGA, filled square—glucose, filled circle—dry cell weight. The error bars represent the standard deviation of three independent replicates.

The time course of 2KGA production and consumption by *P. plecoglossicida* JUIM01, when the initial glucose concentration of fermentation medium was 14 g/L, is shown in Figure 2a. At 10 h of fermentation, 10.5 g/L of 2KGA was produced, and almost all the glucose was consumed. After glucose depletion, the strain started to consume the product 2KGA. This phenomenon is very similar to that described by Fuhrer et al. [13].

Next, another 2KGA fermentation by *P. plecoglossicida* JUIM01 was performed. In this fermentation process, 14 g/L of glucose was fed into fermentation broth after glucose was exhausted. As shown in Figure 2b, after the supplementation of glucose, the strain continued to produce 2KGA by using glucose; only when the glucose in fermentation broth was exhausted did the strain start to consume the product 2KGA.

Then, the experiment of 2KGA supplementation during the fermentation process was performed. During the fermentation process, 15 g/L of 2KGA was fed into the fermentation broth before glucose was exhausted. As shown in Figure 2c, after supplementing 2KGA, the strain continued to produce 2KGA by using the residual glucose in fermentation broth; similarly, only when the glucose was exhausted did the strain start to consume the product 2KGA.

As mentioned above, free-living bacteria usually have a versatile metabolism and can selectively assimilate a preferred compound through cell regulatory effects of carbon catabolite repression of gene expression [15,16]. Until now, there is almost no detailed research on the carbon catabolite repression of 2KGA metabolism by glucose in *Pseudomonas* strain, and there is no research on the relationship between glucose and 2KGA metabolism in *P. plecoglossicida* strain. The results of these three experiments of 2KGA fermentation by *P. plecoglossicida* JUIM01 at low initial glucose concentration indicate that *P. plecoglossicida* JUIM01 possesses carbon catabolite repression of 2KGA metabolism by glucose. Only after the glucose was exhausted did the strain start to assimilate 2KGA for cell growth and maintenance.

### 2.2. Transcription and Proteomics Studies on 2KGA Consumption by P. plecoglossicida JUIM01

The transcription levels of *gcd*, *gad*, *kguK*, *kguD*, *glk*, and *gnuK* genes were studied during 2KGA fermentation by *P. plecoglossicida* JUIM01 with initial glucose concentration of 14 g/L, and without glucose and 2KGA supplementation. The results are shown in Table 1. In the initial stage of fermentation, 2KGA was produced by *P. plecoglossicida* JUIM01 using glucose (Figure 2a). Meanwhile, the transcription levels of *gcd* and *gad* genes for 2KGA biosynthesis were increased before 12 h of fermentation process, especially the *gcd* gene. In the later phase of fermentation, all the glucose was consumed, and the strain started to consume the product 2KGA (Figure 2a). In the meantime, the transcription levels of *kguK* and *kguD* genes for 2KGA metabolism were increased significantly. The transcription levels of *glk* and *gnuK* genes for glucose and gluconate metabolism, respectively, showed no remarkable change (Table 1). The transcription analysis of 2KGA biosynthesis and metabolism genes indicates that *P. plecoglossicida* JUIM01 possesses carbon catabolite repression. The transcriptional expression of genes in *P. plecoglossicida* JUIM01 required for the degradation of 2KGA was repressed until glucose was depleted.

For proteomics analysis, whole-cell protein extraction was performed at the middle stages of 2KGA production phase and consumption phase, during 2KGA fermentation by *P. plecoglossicida* JUIM01 with the initial glucose concentration of 14 g/L, and without glucose and 2KGA supplementation. In the 2KGA consumption phase, 11 proteins were upregulated in expression and 23 proteins were downregulated in expression, compared to those in 2KGA production phase (Figure S1, Tables S1 and S2). In 2KGA consumption phase, 2 ATP-binding cassette transporters (protein spots 5419 and 5423) were upregulated in expression, and it is speculated that the cell needed to assimilate amino acids for cell growth and maintenance after all the glucose was consumed. Several proteins (protein spots 2107, 5220, 5311, and 6423), participating in cellular metabolic reactions, were upregulated in expression; this is probably due to the change of cellular metabolism when 2KGA was used as a substrate by the cell (Table S1). In the 2KGA consumption phase, the proteins participating in biosynthesis of amino acids (protein spots 6518 and 6519), nucleotides (protein spot 6714), and fatty acids (protein spot 6514, 7113, and 7612), were downregulated in expression (Table S2). The protein 1034 is a component of ribosome, and the protein 6420 is used for peptidoglycan biosynthesis; these two proteins participate in protein synthesis and cell wall formation, and their expression levels were downregulated (Table S2). In the 2KGA consumption phase, many proteins participating in cell growth were downregulated in expression; thus, the cell growth rate was decreased (Figure 2a).

The transcription analysis in 2KGA fermentation by *P. plecoglossicida* JUIM01 revealed that the transcription levels of genes for 2KGA metabolism were significantly increased (more than 10 times) after glucose depletion. However, the proteomics study by 2-DE could not detect the proteins participating in 2KGA metabolism and their changes in expression. This was perhaps a result of the limitations of protein fractionation by 2-DE. The proteins participating in 2KGA metabolism could not be isolated and identified in the process [19]. Besides, the gene expression variation at translation is more conserved than transcription, mostly due to the buffering effect of translational regulation for the transcriptional divergence [20]. Although there were changes in expression of the proteins participating in 2KGA metabolism, the changes were less significant. In the future, the quantitative iTRAQ LC-MS/MS proteomics can be used to perform a more global detection and functional inference of proteins involved in 2KGA metabolism and carbon catabolite repression of 2KGA metabolism by glucose [21,22].

### 2.3. Attenuation of 2KGA Consumption by Increasing the Supply of Glucose

**Figure 3:**
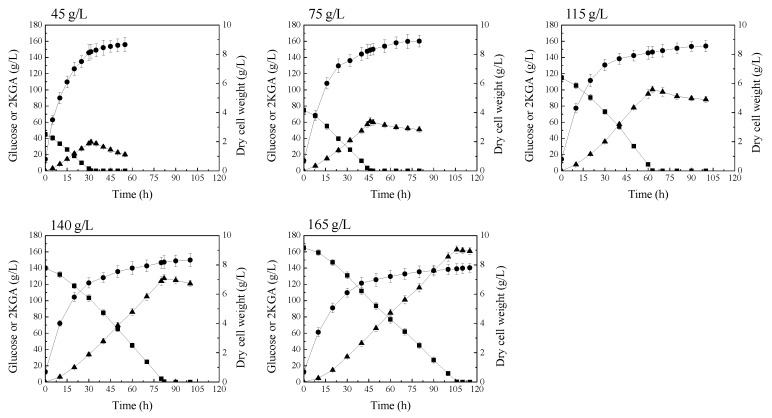
Attenuation of 2KGA consumption by increasing the supply of glucose. Signal denotes: filled triangle—2KGA, filled square—glucose, filled circle—dry cell weight. The error bars represent the standard deviation of three independent replicates.

The above studies indicated that *P. plecoglossicida* JUIM01 possesses carbon catabolite repression, and glucose is the preferred carbon source compared to 2KGA. Glucose in culture broth can repress the 2KGA metabolism by *P. plecoglossicida* JUIM01. In order to attenuate 2KGA consumption by *P. plecoglossicida* JUIM01, the initial glucose concentration of fermentation medium was increased. Time courses of 2KGA production and consumption at different initial glucose concentrations by *P. plecoglossicida* JUIM01 are shown in Figure 3. These results showed that increasing the initial glucose concentration of the fermentation medium was effective in attenuating 2KGA consumption after glucose depletion. This is probably because the cell vitality of *P. plecoglossicida* JUIM01 declined after a long time of cultivation, and the vitality decline became more and more serious, along with the prolonged cultivation process. More importantly, the 2KGA yield from glucose was enhanced, along with the increased initial glucose concentration of fermentation medium, when 2KGA concentration reached the highest point (Table 2). This is mostly because the proportion of glucose used for biomass or cell growth decreased and, thus, the proportion of glucose used for 2KGA production was enhanced with the increased glucose supply (Table 2). However, excessive glucose concentration in the fermentation medium had an adverse effect on cell growth and 2KGA production rate (Figure 3).

### 2.4. Fed-Batch Fermentation of P. plecoglossicida JUIM01 for 2KGA Production

**Figure 4:**
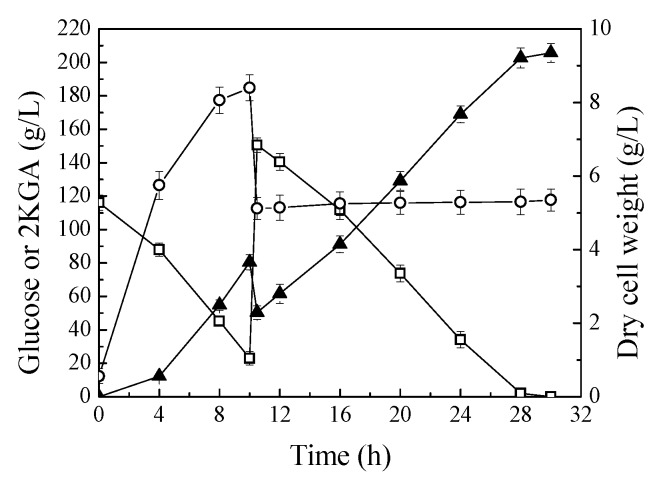
Fed-batch fermentation of *P. plecoglossicida* JUIM01 for 2KGA production. Signal denotes: filled triangle—2KGA, empty square—glucose, empty circle—dry cell weight. The error bars represent the standard deviation of three independent replicates.

The production performance of *P. plecoglossicida* JUIM01 was investigated in fed-batch process. Figure 4 shows the time profiles of fed-batch fermentations in 30 L bioreactors. Fed-batch fermentation of *P. plecoglossicida* JUIM01 resulted in 205.67 g/L of 2KGA with a productivity of 6.86 g/L/h and yield of 0.953 g/g glucose. The final concentration and productivity of 2KGA in fed-batch fermentation process were significantly increased compared to batch fermentation in shake flasks. This is because the biosynthesis of 2KGA is an oxidation process of glucose, and the oxygen supply of fermentation in a stirred fermenter is more abundant than that of fermentation in shake flasks [9,11]. In addition, fed-batch fermentation can attenuate the adverse effects of excessive glucose concentration, in the fermentation medium, on cell growth. Under optimum conditions, *K. pneumoniae* could produce 186 g/L of 2KGA with a productivity of 7.15 g/L/h, and yield 1.05 g/g of glucose using a two-stage fermentation strategy, and the productivity and yield from glucose is the highest in the research of 2KGA production by direct fermentation [6]. Therefore, *P. plecoglossicida* JUIM01 has great competitiveness and practicability for 2KGA production by direct fermentation.

Metabolic engineering has proven to be important in developing competitive microbial strains for the production of diverse chemicals [23,24,25,26]. Until now, almost all of the metabolic engineering work for 2KGA production were focused on the 2KGA synthesis pathway, including glucose dehydrogenase and gluconate 2-dehydrogenase [8,9,27]. In the future, studies on the detailed relationship between 2KGA synthesis, glucose metabolism, and cell growth and maintenance, will provide valuable information for 2KGA production. On this basis, systems metabolic engineering, for optimizing the relationship among 2KGA synthesis, glucose metabolism, and cell growth and maintenance, will further increase the 2KGA production efficiency.

## 3. Materials and Methods

### 3.1. Microorganism and Cultivation Conditions

*P. plecoglossicida* JUIM01 strain, that was screened and stored in our laboratory, is an important industrial 2KGA producer, and has been deposited in the China General Microbiological Culture Collection Center (CGMCC) under collection number CGMCC No. 7150 [7]. The strain was maintained on slant medium containing peptone 10 g/L, beef extract 5 g/L, NaCl 5 g/L, and agar 20 g/L. The strain from agar slants was inoculated in 50 mL seed medium containing glucose 20 g/L, corn steep liquor 5 g/L, urea 2 g/L, KH_2_PO_4_ 2 g/L, MgSO_4_·7H_2_O 0.5 g/L, CaCO_3_ 1 g/L, and cultured at 30 °C for 18 h in 500 mL shake flasks with a rotational speed of 265 rpm. The seed culture (5 mL) was transferred into 500 mL shake flasks containing 50 mL fermentation medium, and cultured at 30 °C with a rotational speed of 265 rpm. The fermentation medium contained corn steep liquor 5 g/L, urea 2 g/L, KH_2_PO_4_ 2 g/L, MgSO_4_·7H_2_O 0.5 g/L, the glucose concentration of the fermentation medium was adjusted to the desired levels according to the experimental design, and CaCO_3_ was added for balancing the pH from 5.0 to 6.5, and its concentration was a quarter of the glucose concentration.

For 2KGA fed-batch fermentation, the experiments were carried out at 30 L lab scale in a mechanically stirred fermenter (GRJ-30D, Green Bio-engineering Co. Ltd., Zhenjiang, China). The seed culture (1.5 L) was obtained as described above. The seed culture was transferred into the 30 L stirred fermenter containing 15 L fermentation medium, consisting of glucose 115 g/L, corn steep liquor 5 g/L, urea 2 g/L, KH_2_PO_4_ 2 g/L, MgSO_4_·7H_2_O 0.5 g/L, CaCO_3_ 35 g/L, and antifoaming agent 0.2 g/L. The feed medium (9 L) consisting of glucose 350 g/L and CaCO_3_ 100 g/L, was fed into the fermenter when the residual concentration of glucose went below 20–25 g/L. The fed-batch fermentations were performed at 30 °C, the agitation speed was controlled at 440 rpm, and the air flow rate was maintained at 1.5 vvm.

### 3.2. Analytical Methods

Dry cell weight (DCW) was determined from a calibration curve of known DCW and the corresponding optical density at 650 nm (DCW = 0.44 × OD_650_, g/L) using a spectrophotometer (Biospec-1601 spectrophotometer, Shimadzu) [3]. For quantification of substrate consumption and product formation, samples of the culture were harvested and spun down (10,000*g*, 5 min, and 4 °C). Glucose concentration was determined using a Biosensor Analyzer (Biology Institute of Shandong Academy of Sciences, Jinan, China). The concentration of 2KGA was determined and calculated on the basis of glucose concentration, using a polarimetry method [3,7]. All experiments were carried out in triplicates, independently, and the results were the average of three replicate experiments.

### 3.3. Transcriptional Analysis

Total RNA was extracted from *P. plecoglossicida* JUIM01 cells at different time points of the cultivation using the RNAiso Plus reagent (Takara, Dalian, China). cDNA was synthesized using a PrimeScript RT reagent kit (Takara, Dalian, China). Real-time PCR (RT-PCR) was performed as described by Zhang et al. 2013 [28]. Normalization of the results was performed by using *rpoD* as the housekeeping gene, and the relative abundance of *rpoD* gene was used as the internal standard [29]. The primers used for RT-PCR analysis are listed in Table 3. All the experiments were carried out in triplicates independently, and the results were the average of three replicate experiments.

### 3.4. Whole-Cell Protein Extraction, Two-Dimensional Gel Electrophoresis, and Protein Identification

Sample preparation was performed at the middle stages of 2KGA production phase and the consumption phase. Cells were harvested by centrifugation (10,000 × *g*, 1 min, and 4 °C) and immediately frozen in liquid nitrogen. The whole-cell protein extraction was performed according to reference [30]. Proteins were separated by two-dimensional gel electrophoresis (2-DE). For isoelectric focusing, 1 mg of proteins were diluted in the immobilized pH gradient (IPG) strip rehydration buffer (450 μL). The protein solution was loaded on 24 cm IPG strips (GE Healthcare) that provided a linear gradient from pH 3 to 10, according to the manufacturer’s instructions. Isoelectric focusing and sodium dodecyl sulfate (SDS)-polyacrylamide electrophoresis were carried out according to references [30,31]. Stained 2-DE gels were scanned using an ImageScanner III (GE Healthcare) and analyzed by PDQuest 2-D Analysis Software (Bio-Rad). Protein spots of interest were cut from the gels and trypsin digested in-gel, as described, and identified by MALDI-TOF/TOF tandem MS using an AB SCIEX 5800 MALDI TOF/TOF mass spectrometer (AB SCIEX, USA). MS data were analyzed using the MASCOT 2.1.0 program (Matrix Science, MA), as described [30,31,32].

## 4. Conclusions

In this paper, we found the 2KGA-producing strain, *P. plecoglossicida* JUIM01, could consume 2KGA after glucose depletion during the fermentation process. Glucose and 2KGA supplementation experiments, and transcription analysis of 2KGA biosynthesis and metabolism genes, indicated that *P. plecoglossicida* JUIM01 exhibits carbon catabolite repression of 2KGA metabolism by glucose. The consumption of 2KGA by *P. plecoglossicida* JUIM01 was attenuated by supplement of sufficient glucose, and the final concentration and productivity of 2KGA could reach 205.67 g/L and 6.86 g/L/h, respectively, during fed-batch fermentation of *P. plecoglossicida* JUIM01. Further systematic studies on the consumption of 2KGA by *P. plecoglossicida* JUIM01, using genome-wide metabolic model in combination with integrated omics analysis, can provide important guidance for the industrial fermentation production of 2KGA.

## Figures and Tables

**Table 1:** Relative transcriptional levels of genes for 2KGA biosynthesis and metabolism at different fermentation phases.

Time (h)	Relative Transcriptional Levels					
	*gcd*	*gad*	*kguK*	*kguD*	*glk*	*gnuK*
4	1.0 ± 0.2	1.0 ± 0.3	1.0 ± 0.2	1.0 ± 0.2	1.0 ± 0.1	1.0 ± 0.2
8	1.1 ± 0.3	1.2 ± 0.2	1.1 ± 0.1	1.6 ± 0.3	0.5 ± 0.2	0.7 ± 0.2
12	6.2 ± 1.5	1.7 ± 0.4	2.0 ± 0.6	4.5 ± 1.4	3.0 ± 0.9	0.9 ± 0.4
16	5.0 ± 2.1	1.5 ± 0.3	14.2 ± 2.5	11.8 ± 2.0	1.7 ± 0.4	1.6 ± 0.5

**Table 2:** 2KGA batch fermentation parameters at different initial glucose concentrations when the 2KGA concentration reached the highest point.

Glucose (g/L)	2KGA Production (g/L)	2KGA Yield on Glucose (g/g)	Productivity (g/L/h)	Biomass (g/L)	Biomass Yield on Glucose (g/g)
45	35.6 ± 3.0	0.79 ± 0.07	1.13 ± 0.09	8.2	0.18
75	61.2 ± 4.8	0.82 ± 0.06	1.33 ± 0.10	8.2	0.11
115	100.4 ± 4.3	0.87 ± 0.04	1.58 ± 0.07	8.1	0.07
140	127.3 ± 4.6	0.91 ± 0.03	1.55 ± 0.06	8.0	0.06
165	162.7 ± 4.8	0.99 ± 0.03	1.53 ± 0.04	7.7	0.05

**Table 3:** Primers used for RT-PCR analysis.

Name	Sequence (5′→3′)
*rpoD*F	GATTCGTCAGGCGATCAC
*rpoD*R	AATACGGTTGAGTTGTTGA
*gcd*F	ACCAGTACCTGCGTGCCTAT
*gcd*R	CCTTGCCGGTGTAGGTCAT
*gad*F	TTTCATGGATTGGGTGGAAC
*gad*R	CGCATCGACTTTCTTCATCA
*kguK*F	GCGACCCGCAAGTGGAATAC
*kguK*R	GAAGGAGATGCTGCGACCGT
*kguD*F	CCGAAACCACTGCCGACACC
*kguD*R	ACGATGCCCAGCGTCTTGC
*glk*F	CTGCATGAGCGGGTATTTC
*glk*R	ATGATCCAACGCCTGCTG
*gnuK*F	GTTCGGACTGGCTACTGATACC
*gnuK*R	ACCGCCAAAGCCGTCCT

## Supplementary Materials

The supplementary materials are available online.

## References

[1] Sun W.J., Liu C.F., Yu L., Cui F.J., Zhou Q., Yu S.L., Sun L. (2012). A novel bacteriophage KSL-1 of 2-Keto-gluconic acid producer *Pseudomonas fluorescens* K1005: Isolation, characterization and its remedial action. BMC Microbiol..

[2] Sun W., Xiao F., Wei Z., Cui F., Yu L., Yu S., Zhou Q. (2015). Non-sterile and buffer-free bioconversion of glucose to 2-keto-gluconic acid by using *Pseudomonas fluorescens* AR4 free resting cells. Process Biochem..

[3] Sun W.J., Zhou Y.Z., Zhou Q., Cui F.J., Yu S.L., Sun L. (2012). Semi-continuous production of 2-keto-gluconic acid by *Pseudomonas fluorescens* AR4 from rice starch hydrolysate. Bioresour. Technol..

[4] Kiefler I., Bringer S., Bott M. (2017). Metabolic engineering of *Gluconobacter oxydans* 621H for increased biomass yield. Appl. Microbiol. Biotechnol..

[5] Krajewski V., Simic P., Mouncey N.J., Bringer S., Sahm H., Bott M. (2010). Metabolic engineering of *Gluconobacter oxydans* for improved growth rate and growth yield on glucose by elimination of gluconate formation. Appl. Environ. Microbiol..

[6] Sun Y., Wei D., Shi J., Mojović L., Han Z., Hao J. (2014). Two-stage fermentation for 2-Ketogluconic acid production by *Klebsiella pneumoniae*. J. Microbiol. Biotechnol..

[7] Sun W., Wang Q., Luan F., Man Z., Cui F., Qi X. (2018). The role of kguT gene in 2-ketogluconate-producing *Pseudomonas plecoglossicida* JUIM01. Appl. Biochem. Biotechnol..

[8] Yi X., Li T., Wang B., Liu J., Du H., Feng H. (2014). Production of 2-Keto-D-gluconic acid by metabolically engineered *Gluconobacter suboxydans*. China Biotechnol..

[9] Li K., Mao X., Liu L., Lin J., Sun M., Wei D., Yang S. (2016). Overexpression of membrane-bound gluconate-2-dehydrogenase to enhance the production of 2-keto-d-gluconic acid by *Gluconobacter oxydans*. Microb. Cell Fact..

[10] del Castillo T., Ramos J.L., Rodríguez-Herva J.J., Fuhrer T., Sauer U., Duque E. (2007). Convergent peripheral pathways catalyze initial glucose catabolism in *Pseudomonas putida*: Genomic and flux analysis. J. Bacteriol..

[11] Nikel P.I., Chavarría M., Fuhrer T., Sauer U., de Lorenzo V. (2015). *Pseudomonas putida* KT2440 strain metabolizes glucose through a cycle formed by enzymes of the Entner-Doudoroff, Embden-Meyerhof-Parnas, and Pentose Phosphate Pathways. J. Biol. Chem..

[12] Kim J., Jeon C.O., Park W. (2008). Dual regulation of *zwf-1* by both 2-keto-3-deoxy-6-phosphogluconate and oxidative stress in *Pseudomonas putida*. Microbiology.

[13] Fuhrer T., Fischer E., Sauer U. (2005). Experimental identification and quantification of glucose metabolism in seven bacterial species. J. Bacteriol..

[14] del Castillo T., Ramos J.L. (2007). Simultaneous catabolite repression between glucose and toluene metabolism in *Pseudomonas putida* is channeled through different signaling pathways. J. Bacteriol..

[15] Rojo F. (2010). Carbon catabolite repression in *Pseudomonas*: Optimizing metabolic versatility and interactions with the environment. FEMS Microbiol. Rev..

[16] Sonnleitner E., Abdou L., Haas D. (2009). Small RNA as global regulator of carbon catabolite repression in *Pseudomonas aeruginosa*. Proc. Natl. Acad. Sci. USA.

[17] Kim J., Yeom J., Jeon C.O., Park W. (2009). Intracellular 2-keto-3-deoxy-6-phosphogluconate is the signal for carbon catabolite repression of phenylacetic acid metabolism in *Pseudomonas putida* KT2440. Microbiology.

[18] Basu A., Phale P.S. (2006). Inducible uptake and metabolism of glucose by the phosphorylative pathway in *Pseudomonas putida* CSV86. FEMS Microbiol. Lett..

[19] Liang C.R.M.Y., Leow C.K., Neo J.C.H., Tan G.S., Lo S.L., Lim J.W.E., Seow T.K., Lai P.B.S., Chung M.C.M. (2005). Proteome analysis of human hepatocellular carcinoma tissues by two-dimensional difference gel electrophoresis and mass spectrometry. Proteomics.

[20] Wang Z., Sun X., Zhao Y., Guo X., Jiang H., Li H., Gu Z. (2015). Evolution of gene regulation during transcription and translation. Genome Biol. Evol..

[21] Qiao J., Shao M., Chen L., Wang J., Wu G., Tian X., Liu J., Huang S., Zhang W. (2013). Systematic characterization of hypothetical proteins in *Synechocystis* sp. PCC 6803 reveals proteins functionally relevant to stress responses. Gene.

[22] Qiao J., Wang J., Chen L., Tian X., Huang S., Ren X., Zhang W. (2012). Quantitative iTRAQ LC-MS/MS proteomics reveals metabolic responses to biofuel ethanol in cyanobacterial *Synechocystis* sp. PCC 6803. J. Proteome Res..

[23] Man Z., Rao Z., Xu M., Guo J., Yang T., Zhang X., Xu Z. (2016). Improvement of the intracellular environment for enhancing L-arginine production of *Corynebacterium glutamicum* by inactivation of H_2_O_2_-forming flavin reductases and optimization of ATP supply. Metab. Eng..

[24] Zhang X., Zhang R., Bao T., Rao Z., Yang T., Xu M., Xu Z., Li H., Yang S. (2014). The rebalanced pathway significantly enhances acetoin production by disruption of acetoin reductase gene and moderate-expression of a new water-forming NADH oxidase in *Bacillus subtilis*. Metab. Eng..

[25] Man Z., Xu M., Rao Z., Guo J., Yang T., Zhang X., Xu Z. (2016). Systems pathway engineering of *Corynebacterium crenatum* for improved L-arginine production. Sci. Rep..

[26] Guo J., Man Z., Rao Z., Xu M., Yang T., Zhang X., Xu Z. (2017). Improvement of the ammonia assimilation for enhancing L-arginine production of Corynebacterium crenatum. J. Ind. Microbiol. Biotechnol..

[27] Merfort M., Herrmann U., Ha S.W., Elfari M., Bringer-Meyer S., Görisch H., Sahm H. (2006). Modification of the membrane-bound glucose oxidation system in *Gluconobacter oxydans* significantly increases gluconate and 5-keto-D-gluconic acid accumulation. Biotechnol. J..

[28] Zhang X., Zhang R., Bao T., Yang T., Xu M., Li H., Xu Z., Rao Z. (2013). Moderate expression of the transcriptional regulator ALsR enhances acetoin production by *Bacillus subtilis*. J. Ind. Microbiol. Biotechnol..

[29] Kremmydas G.F., Tampakaki A.P., Georgakopoulos D.G. (2013). Characterization of the biocontrol activity of *Pseudomonas fluorescens* strain X reveals novel genes regulated by glucose. PLoS ONE.

[30] Gnoni A., Lippolis R., Zanotti F., Papa S., Palese L.L. (2007). A two-dimensional electrophoresis and mass spectrometry protein analysis of the antibiotic producer *Nonomuraea* sp. ATCC 39727 in different growth conditions. FEMS Microbiol. Lett..

[31] Zhou J., Wang K., Xu S., Wu J., Liu P., Du G., Li J., Chen J. (2015). Identification of membrane proteins associated with phenylpropanoid tolerance and transport in *Escherichia coli* BL21. J. Proteomics.

[32] Jiang W., Du B., Chi Z., Ma L., Wang S., Zhang X., Wu W., Wang X., Xu G., Guo C. (2007). Preliminary explorations of the role of mitochondrial proteins in refractory epilepsy: Some findings from comparative proteomics. J. Neurosci. Res..

