# Supplementary material for: Enhancing 2-Ketogluconate Production of Pseudomonas plecoglossicida JUIM01 by Maintaining the Carbon Catabolite Repression of 2-Ketogluconate Metabolism
Source: Molecules. 2018 Oct 13;23(10):2629. doi: 10.3390/molecules23102629 (PMC6222622; doi:10.3390/molecules23102629)
Supplement: Supplementary file 1 [file molecules-23-02629-s001.pdf]

Supplementary Material

Enhancing 2-ketogluconate Production of *Pseudomonas plecoglossicida* JUIM01 by Maintaining the Carbon Catabolite Repression of 2-ketogluconate Metabolism

Wenjing Sun [1,2,*], Tjahjasari Alexander [1], Zaiwei Man [1,*], Fangfang Xiao [1], Fengjie Cui [1,2] and Xianghui Qi [1]

[1] School of Food and Biological Engineering, Jiangsu University, Zhenjiang 212013, China; 1000003523@ujs.edu.cn (T.A.); 1000004769@ujs.edu.cn (F.X.); 1000003286@ujs.edu.cn (F.C.); 1000003420@ujs.edu.cn (X.Q.)

[2] Parchn Sodium Isovitamin C Co. Ltd, Dexing, 334221, China

* Correspondence: juswj@ujs.edu.cn (W.S.); qiangni.well@163.com (Z.M.)

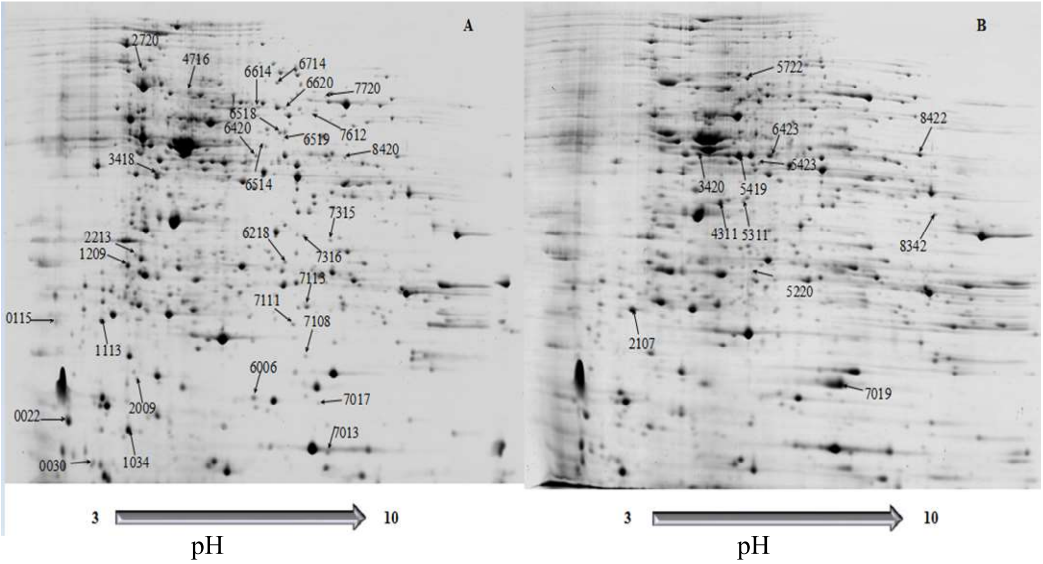

Figure S1. 2DE analysis of protein expression in *P. plecoglossicida* JUIM01 at the middle stages of 2-KGA production phase (**A**) and consumption phase (**B**), during the 2KGA fermentation with the initial glucose concentration of 14 g/L and without glucose and 2KGA supplement.

Table S1. Identification of proteins up-regulated in expression in 2-KGA consumption phase.

Spot No.	Accession No.	Protein Name	Gene Symbol and Functional Annotation	Organism	Mw/pI	No. of Peptides Matched	Sequence Coverage	MASCOT Score [a]	Fold Change
3420	gi∣515086137	Spermidine/ putrescine ABC transporter substrate-binding protein	*potD*	*Pseudomonas monteilii*	37804/5.44	5	18%	350	7.8600
5419	gi∣495364857	ABC-type branched-chain amino acid transport system, periplasmic component	*livJ* cell envelope, external encapsulating structure	*Pseudomonas sp.* GM84	40325/5.93	6	20%	686	2.0065
5423	gi∣496887707	branched-chain amino acid ABC transporter periplasmic amino acid-binding protein	*livJ* cell envelope, external encapsulating structure	*Pseudomonas putida*	40225/5.74	3	13%	97	1.7990
4311	gi∣167035543	extracellular solute-binding protein	*yhdw* cell envelope, external encapsulating structure	*Pseudomonas putida* GB-1	36672/5.82	9	36%	875	2.6699
6423	gi∣447918323	Ketol-acid reductoisomerase	*ilvC* organic acid metabolic process, single-organism biosynthetic process, small molecule metabolic process, organonitrogen compound metabolic process, cytoplasm	*Pseudomonas poae* RE*1-1-14	36418/5.48	2	8%	101	1.5927

5722	gi|167031247	hypothetical protein Dipeptidase	*PputGB1_0229*	*Pseudomonas putida* GB-1	62469/5.66	2	5%	237	1.5913
5220	gi|26989841	3-oxoacid CoA-transferase subunit A	*scoA*	*Pseudomonas putida* KT2440	25223/5.44	9	45%	771	7.0543
2107	gi|26987276	inorganic pyrophosphatase	*ppa* cytoplasm, Oxidative phosphorylation	*Pseudomonas putida* KT2440	19177/4.77	1	6%	65	3.1488
5311	gi|26989058	2-methylisocitrate lyase	*prpB* organic acid metabolic process, small molecule metabolic process, cellular lipid metabolic process, lipid metabolic process, Propanoate metabolism,	*Pseudomonas putida* KT2440	31962/5.45	2	8%	93	2.5975
8422	gi|495262346	Uncharacterized protein	*PMI28_02953*	*Pseudomonas sp.* GM48	44393/6.27	7	25%	572	1.9237
8342	gi|26990659	Periplasmic binding protein, putative	*PP_3954*	*Pseudomonas putida* KT2440	33219/8.86	2	9%	172	18.7612

[a] Individual ion scores > 59 indicate identity or extensive homology.

Table S2. Identification of proteins down-regulated in expression in 2-KGA consumption phase.

Spot No.	Accession No.	Protein Name	Gene Symbol and Functional Annotation	Organism	Mw/pI	No. of Peptides Matched	Sequence Coverage	MASCOT Score[a]	Fold Change
4716	gi∣496892991	phosphoenolpyruvate carboxykinase [ATP]	*pckA* single-organism biosynthetic process, nucleoside phosphate binding, nucleotide binding	*Pseudomonas putida*	55920/5.24	2	4%	118	0.1745
7720	gi∣170724230	succinate CoA transferase	*cat1*	*Pseudomonas putida* W619	54048/5.64	6	13%	239	0.1037
6519	gi∣557225030	Glutamate dehydrogenase	*gdhA* organic acid metabolic process, single-organism biosynthetic process, small molecule metabolic process, organonitrogen compound metabolic process, oxidoreductase activity, Alanine, aspartate and glutamate metabolism	*Pseudomonas sp.* VLB120	48935/6.22	7	20%	377	0.3378
6518	gi∣104780671	3-phosphoshikimate 1-carboxyvinyltransferase	*aroA* organic acid metabolic process, single-organism biosynthetic process, small molecule metabolic process, organonitrogen compound metabolic	*Pseudomonas entomophila* L48	79605/5.85	6	8%	374	0.5326

			process, transferase activity						
6620	gi|512620677	methylmalonate-semialdehyde dehydrogenase	*mmsA*	*Pseudomonas resinovorans* NBRC 106553	53829/5.64	4	12%	269	0.4275
6714	gi|557224572	CTP synthetase	*pyrG* single-organism biosynthetic process, organonitrogen compound metabolic process, cytoplasm, ligase activity forming carbon-nitrogen bonds, nucleoside phosphate binding, nucleotide binding	*Pseudomonas sp.* VLB120	59829/5.56	8	20%	743	0.5732
6514	gi|170720713	3-oxoacyl-(acyl carrier protein) synthase II	*fabF* organic acid metabolic process, single-organism biosynthetic process, small molecule metabolic process, cellular lipid metabolic process, lipid metabolic process, cytoplasm, fatty acid biosynthesis, biotin metabolism,	*Pseudomonas putida* W619	43377/5.60	5	14%	283	0.4328

7113	gi|26988643	3-ketoacyl-ACP reductase	*fabG* organic acid metabolic process, single-organism biosynthetic process, cellular lipid metabolic process, lipid metabolic process, organonitrogen compound metabolic process, cofactor metabolic process, fatty acid biosynthesis, biotin metabolism, biosynthesis of unsaturated fatty acids	*Pseudomonas putida* KT2440	25454/5.94	7	31%	508	0.5897
7612	gi|26987296	acetyl-CoA carboxylase biotin carboxylase subunit	*accC* organic acid metabolic process, single-organism biosynthetic process, small molecule metabolic process, cellular lipid metabolic process, lipid metabolic process, thioester metabolic process, organonitrogen compound metabolic process, cofactor metabolic process, fatty acid biosynthesis, propanoate metabolism	*Pseudomonas putida* KT2440	49456/6.09	8	24%	585	0.4062
8420	gi|511760925	4-aminobutyrate aminotransferase	*gabT* organic acid metabolic process, small molecule metabolic process,	*Pseudomonas plecoglossicida*	45238/5.87	8	25%	571	0.4217

			organonitrogen compound metabolic process, propanoate metabolism, butanoate metabolism, alanine, aspartate and glutamate metabolism						
6218	gi|15598367	3-demethylubiquinone-9 3-methyltransferase	*ubiG*<br>single-organism biosynthetic process, small molecule metabolic process, cofactor metabolic process,	*Pseudomonas aeruginosa* PAO1	26014/5.91	3	11%	104	0.3581
2720	gi|167035479	60 kDa chaperonin	*groL*<br>cytoplasm, nucleoside phosphate binding, nucleotide binding	*Pseudomonas putida* GB-1	56665/4.96	10	26%	1096	0.2746
22	gi|151243	60 kDa chaperonin	*groL*<br>cytoplasm, nucleoside phosphate binding, nucleotide binding	*Pseudomonas aeruginosa*	57252/5.09	1	3%	83	0.5876
6006	gi|167035043	cyclophilin type peptidyl-prolyl cis-trans isomerase	*ppiA*<br>cell envelope, external encapsulating structure	*Pseudomonas putida* GB-1	20275/8.64	5	36%	441	0.6454
1209	gi|26991896	isoprenoid biosynthesis protein with amidotransferase-like domain	*elbB*<br>single-organism biosynthetic process, cellular lipid metabolic process, lipid metabolic process	*Pseudomonas putida* KT2440	23775/5.19	3	16%	190	0.5342

2213	gi|511759234	hydrolase alpha/beta fold family protein	*mhpC* organic acid metabolic process, small molecule metabolic process, response to xenobiotic stimulus, hydrolase activity acting on acid carbon-carbon bonds, phenylalanine metabolism	*Pseudomonas plecoglossicida*	30159/5.31	2	5%	111	0.3581
3418	gi|104780339	xenobiotic reductase B	*xenB*	*Pseudomonas entomophila* L48	37740/5.42	7	24%	631	0.5206
7111	gi|557224377	nitroreductase	*PVLB_18420*	*Pseudomonas sp.* VLB120	21990/5.96	2	13%	184	0.5275
6614	gi|77461949	F0F1 ATP synthase subunit alpha	*atpA* single-organism biosynthetic process, small molecule metabolic process, organonitrogen compound metabolic process, proton-transporting two-sector ATPase complex catalytic domain, proton-transporting ATP synthase complex, nucleoside phosphate binding, nucleotide binding, oxidative phosphorylation	*Pseudomonas fluorescens* Pf0-1	55459/5.39	3	8%	157	0.4946

115	gi|518238341	chemotaxis protein CheY	*cheY*	*Pseudomonas psychrophila*	20729/5.25	4	30%	252	0.1767
1034	gi|104783860	30S ribosomal protein S6	*rpsF*	*Pseudomonas entomophila* L48	16386/4.94	4	39%	244	0.3193
7108	gi|32469937	single-stranded DNA-binding protein	*ssb*	*Pseudomonas putida*	18357/6.59	2	14%	115	0.6527
6420	gi|557224753	UDP-N-acetylglucosamine 1-carboxyvinyltransferase	*murA* single-organism biosynthetic process, small molecule metabolic process, organonitrogen compound metabolic process, transferase activity,	*Pseudomonas sp.* VLB120	44901/5.43	7	25%	287	0.3725

[a] Individual ion scores > 59 indicate identity or extensive homology.
